# Involvement of microRNAs as a Response to Phototherapy and Photodynamic Therapy: A Literature Review

**DOI:** 10.3390/antiox10081310

**Published:** 2021-08-20

**Authors:** Francesco Borgia, Paolo Custurone, Lucia Peterle, Giovanni Pioggia, Fabrizio Guarneri, Sebastiano Gangemi

**Affiliations:** 1 School and Operative Unit of Dermatology, Department of Clinical and Experimental Medicine, University of Messina, Via Consolare Valeria—Gazzi, 98125 Messina, Italy; paolo.custurone@gmail.com (P.C.); lucia.peterle@gmail.com (L.P.); fguarneri@unime.it (F.G.); 2 Institute for Biolomedical Research and Innovation (IRIB), National Research Council of Italy (CNR), 98164 Messina, Italy; giovanni.pioggia@irib.cnr.it; 3 School and Operative Unit of Allergy and Clinical Immunology, Department of Clinical and Experimental Medicine, University of Messina, Via Consolare Valeria—Gazzi, 98125 Messina, Italy; sebastiano.gangemi@unime.it

**Keywords:** skin, phototherapy, photodynamic therapy, miRNA, epigenetics, skin tumors, psoriasis, antagomir

## Abstract

The current knowledge about the mechanisms of action of light-based treatments (chiefly photodynamic therapy and phototherapy) in skin diseases leans to the possible involvement of epigenetic and oxidative stress mechanisms. To better understand and exploit, to the fullest, these relatively safe and reproducible treatments, several studies have focused on miRNAs, small non-encoding RNAs (22–24 nucleotides), after light-based treatments. The current narrative review focused on 25 articles. A meta-analysis was not deemed appropriate. The results gather the most recurrent skin-related miRNAs up- or downregulated after light treatment. Five of these, miR-21, -29, -125, -145 and -155, are either the most consistently related to efficacy/resistance to treatment or identified as helpful diagnostic tools. A specific class of miRNAs (angioMIRs) requires further studies. Future treatments and imaging techniques could benefit greatly from the use of antagomirs as a possible co-adjuvant therapy along with light-based treatments.

## 1. Introduction

Thanks to the progress of modern research, previously defined medical conditions seem to be more and more well described at a molecular level. One of the missing links of this complex intertwining of external and internal processes is epigenetics, literally “above genetics”, meaning those processes that link genes and their transcriptional products at a very fine level. One of the latest findings in this regard is the topic of this review, microRNAs (miRNAs), small non-encoding RNAs with a sequence capable of binding to mRNA strings. The genes encoding miRNAs, usually scattered sparsely around the genome, or included in introns and untranslated regions (UTRs) of protein genes, are a class of molecules very well integrated in the cellular processes involving multicellular organisms. The first studies regarding miRNAs and human pathology were directed in the oncology field. Here, research led to an interesting discovery: miRNAs can be tissue-specific and lose that specificity the more the tumor is undifferentiated [1]. Later, other studies extended the field of research regarding the miRNome to other diseases. The skin, one of the main topics of this review, has been thoroughly studied in this sense. The involvement of microRNAs is well established as a mechanism associated with oxidative stress-induced damage, both as a repair mechanism and a proinflammatory one [2], and oxidative stress, particularly in chronic skin diseases treated with light-based therapy, plays a major role in disease activity and therapy response [3,4,5]. The other significant topic of this paper is light-based therapy. More and more different light sources have been used, especially in dermatology, to cure diseases based on the properties of specific wavelengths. The two main therapies dealt with in this review, phototherapy (PT) and photodynamic therapy (PDT), use light sources to bring some therapeutic effects into action. Starting with phototherapy, it uses short wavelengths, in the ultraviolet B spectrum (UVB, usually 311 nm), to produce sub-cellular effects such as the maturation and migration of cells, and the lengthening of the turnover process, such as in keratinocytes. When paired with psoralens, the light source produces ultraviolet A rays, in a procedure called PUVA. It has been used effectively in those skin disorders where the immune system or the keratinocytes do not undergo the usual physiological processes (i.e., psoriasis, parapsoriasis, atopic dermatitis). On the other hand, photodynamic therapy acts as an immune booster and inflammatory stimulus. The main protagonists of this process are unstable substances called photosensitizers. The photosensitizers thus far produced have been subdivided by generation time, spanning from the first dyes such as phthalocyanine through the second generation including the omnipresent aminolevulinic acid to the latest third generation which combines heme-like substances with lipid receptors or monoclonal antibodies to carry the photosensitizer on the desired target [6]. Once the light-sensitive substance reaches its goal, a light source in the infrared spectrum (about 630 nm in wavelength in most cases) indirectly produces the formation of reactive oxygen species (ROS), allowing the killing of the infected/rogue cells and the stimulation of a controlled and self-limiting inflammatory process. The latter therapy has been developed, mostly, for those diseases where cell replication is higher and the self-healing mechanisms of the diseased cells are lacking, such as infective diseases and cancers.

Both these topics of interest have proven to be useful tools on several occasions for a better understanding of the base mechanisms that regulate the human organism and have shown to be promising therapies for the present and the future, but many links are still missing and leave us with questions. In fact, light-based therapy seems to be affecting cells not just with the brute force of damaging or ablating tissues but also by having a crucial role on a genetic and epigenetic scale, since both these aspects of the human and non-human physiology have proven to be dynamic rather than a still picture. Under the light of the above-mentioned affirmations, the aim of this review is as follows:

(1) Organize the current knowledge involving light-based therapy and miRNAs;
(2) Pinpoint the most recurrent and interesting skin-related miRNAs;
(3) Explore possible differences in results for the same disease regarding one light source rather than various light sources.
(4) Propose new applications of the current knowledge for future light-based therapies exploiting miRNAs as targets.

## 2. Materials and Methods

This review was conducted by searching the online database Pubmed. Two different searches were carried out. The first one used the term “phototherapy” along with “miRNA” using the Boolean operator “AND”. The second one used the term “photodynamic therapy” along with “miRNA” using the Boolean operator “AND”. These produced, respectively, 71 and 51 results. Several articles were analyzed and then discarded for they were reviews and books/articles written in any language but English. Subsequently, the remaining articles were chosen after evaluation of the title, abstract and topics dealt with. Duplicates were discarded as well. A total of 25 articles were selected because of their relevance to the purpose of this paper and subdivided into articles regarding photodynamic therapy and ones concerning phototherapy. After the searches, given the great variety of the topics dealt with and the results, it was not deemed appropriate to conduct a systematic review or a meta-analysis due to the low number of articles. Instead, we propose this work in the form of a narrative review. The results are summarized in Table 1.

## 3. Results

Considering the aim of this review, we divided the results into two sections: one regarding articles related to miRNAs as diagnostic tools, and one for miRNAs that are useful as therapeutic means. Regarding photodynamic therapy, the main regime used in these studies was a light source of 630 nm in wavelength and aminolevulinic acid as a photosensitizer. When different from these two items, the photosensitizers were specified in the text. Most of the papers do not provide data about irradiance, light exposure period and total light dose.

The following was found regarding therapeutic means:

Zhang et al. researched, in 2019, miRNA levels upregulated after photoelectric therapy possibly involved in fibrosis. In their study, they found that upon activation by light therapy, the levels of miR-206 were upregulated, promoting the apoptosis and cell migration of human fibroblasts [7].

In 2011, Gu et al. led a study to find the correlation between the levels of miR-21 and -125b after narrowband UVB (NB-UVB) treatment and the levels of p53. The levels of these two miRNAs acted differently. The dosage of miR-21 was decreased and the levels of miR-125 were raised, with a subsequent increase in the levels of p63, a member of the p53 proteins [8].

Still regarding narrowband UVB therapy, Soonthornchai et al. evaluated the levels of miR-155 in immortalized human keratinocytes when paired with a concomitant methotrexate treatment. The results were that after this paired treatment, the levels of caspase-3, a target of miR-155, were significantly lower, leading to a cell cycle arrest in the G0/G1 phase, and led to keratinocyte apoptosis in psoriatic skin samples [9].

In two studies from 2014 and 2016, Chowdari et al. researched the levels of miR-4516 at the lesional level in psoriasis. This miRNA is increased after PUVA therapy and is involved in the downregulation of ubiquitin-conjugating enzyme E2 N (UBE2N), which leads to an increase in p53 at the nuclear level, thus promoting apoptotic processes [10,11].

Wang et al. studied a model in which mesenchymal cells challenged with antagomirs for miR-193 and treated with low doses of irradiation decreased cyclin-dependent kinase 2 (CDK2) and CCND1 expression (which translate in cyclin D1 production), both proteins being related to the cells’ cycle, thus suggesting a possible target to halt the cell cycle [12].

In 2018, a study demonstrated that the combined therapy of vitamin A and fractional laser leads to a boost in skin rejuvenation by increasing the levels of collagen in aged skin. This deposition would be due to the enhanced expression of TGF-beta via the lowering of the miR-29a levels, leading to an activation of the Akt pathway [13].

In 2016, a study by Khori et al. treated mice with an experimental model of breast cancer with low doses of laser therapy, a non-invasive treatment, whilst measuring plasma and intra-tumoral levels of various miRNAs implemented in the cell cycle, such as miR-21, -125 and -155. After 10 treatments of biostimulation via laser treatment, levels of miR-21 and -125 dropped, while those of -155 were upregulated, leading to tumoral loss of mass [14].

On the side of PDT, Hu’s research group studied the effects of acid-mediated sonodynamic therapy using aminolevulinic acid (ALA) on melanoma cells. When treated with this method, the levels of miR-34 were upregulated. This miRNA targets SIRT1, which encodes for sirtuin 1, an inhibitor of p53 acetylation, thus promoting the transcription of p53 which, in a positive loop, increases the expression of miR-34 itself. This effect, combined with the intracellular accumulation of ROS, led to a significant tumoral mass reduction [15].

In 2016, Guo et al., experimenting on human cervical cancer cells, found that PDT therapy leads to an increase in miR-143. This miRNA is involved in the Bcl2/Bax pathway, which regulates the pro- or anti-apoptotic processes. Bcl2 was downregulated, thus increasing the rate of apoptosis of cancerous cells, and upregulating Bax [16]

Moon S. led a study in 2017 where several miRNAs were researched to find potential targets for specific therapies in cases of oral cancer. Of all the miRNAs that were down- or upregulated, the most consistently downregulated of all was miR-145-5p. Given the role of this miRNA in induced apoptosis, a molecule mimicking its effects was developed which, after administration and subsequent PDT treatment, enhanced the intracellular accumulation of ROS and cellular toxicity [17].

A similar study by Fang et al. furthered the hypothesis of an important role of miR-145 in oral cancer cell lines. The authors noticed that an inhibitor of this miRNA, in fact, leads to poorer results after photodynamic therapy in terms of the invasiveness and self-renewal of tumoral cells [18].

Regarding the great chapter of nanosphere-carried treatments, the effects of nanoparticles activated by miR-21 were researched when released inside HeLa cells, after activation with photothermal therapy. The double effect of phototherapy and nanoparticles led to a major killing effect on this subpopulation of cervical cancer cells [19].

Another study regarding cervical cancer cells pinpointed the role of miR-34 as an efficient measurement of photodynamic treatment. The downregulation of this miRNA, in fact, leads to an overexpression of high mobility group box 1 (HMGB1), a protein secreted through exosomes, which seems of vital importance for dendritic cells’ maturation [20].

In a similar experiment, researchers used a combined therapy of melanin carriers binding to mir-145 via a photoacoustic stimulus and photoablation to maximize metastatic cell killing. It is the authors’ belief that a combined therapy could prove useful in the future for better results in tumor suppression [21].

Regarding miRNA inhibitors, a practical therapy proposition was carried out in 2019 by the study group of Wu. In this article, the researchers exploited the inhibition of miR-21 with a graphene-carried inhibitor and treated tumoral cells with phototherapy. The breast cancer cells showed an increase in phosphatase and tensin homolog expression (PTEN) and Bax, thus increasing the apoptosis of tumoral cells and the therapeutic effect of the treatment [22].

Again, using graphene-based nanospheres, another study targeted miR-101. This miRNA, carried inside the cancer cells, activates apoptotic processes and, in order to be released inside the cells, was used a near-infrared thermal light (NIR) [23].

On the same note, Cao et al. combined photothermal therapy and miRNA target therapy exploiting miR-155. In this model, miR-155 acted as a diagnostic tool since its detection inside tumoral cells was underlined by a luciferase-related reaction, and as a therapeutic means, noble metal-based nanoparticles were used to react with a near-infrared light source, thus producing reactive oxygen species capable of cellular toxicity [24].

Finally, another gold-based nanoparticle treatment for theranostic purposes was developed by Qian et al. in 2016, this time targeting miR-21, with comparable results [25].

On the diagnostic side of the results, we provide a review on the following articles:

Mamalis et al. investigated the miRNAs expressed after high-frequency LED red light treatment, showing that the pro-apoptotic ones are upregulated, and the anti-apoptotic ones are downregulated (such as miR-21). Although this study focused on the effects of the light-based treatment on the expressed miRNome, the authors’ conclusion is that such miRNAs require further research, especially when compared to other treatments involving light sources, possibly in an in vivo environment [26].

The prediction of success in terms therapeutic effects was researched by Mcgirt et al. In this study, the levels of miR-191, -223 and -342, three months after treatment, were positively correlated with a clinical response. Although there was no strong explanation to why these specific miRNAs were upregulated after a successful treatment, it is the authors’ opinion that the measurement of their plasma levels could be useful, in the future, for predictive and diagnostic purposes [27].

Ele-refaei, on a similar note, proposed miR-146a as a means of evaluating the clinical response in the case of psoriatic disease. This miRNA was measured both in affected patients and healthy controls, resulting in much higher levels in the case of psoriatic disease. After NB-UVB treatment, it was correlated with the clinical response rate and efficacy. In the conclusion, the authors suggested using this miRNA as a predictive factor for therapeutic efficacy [28].

In a study regarding warts, the miRNome was evaluated after photodynamic treatment. The aim of this model was to propose novel therapeutic targets for the future and pinpoint which miRNA regulates processes that participate in the pathogenesis of warts, such as cell adhesion and cell stress. Given the wide variety of serotypes involved in human papilloma virus (HPV) infection and the fact that this study was led on cultured cells, it is the authors’ opinion that further research is required to make this technology applicable [29].

Li et al. conducted research on the effects of PDT regarding cancer invasiveness and migration. They focused on miR-355, an intron miRNA that seems to be related to PEG1, a gene involved in microtubule organization and the innate immune response. The conclusions were that tumoral cells surviving the PDT treatment are less invasive and lead to a better prognosis for the patients [30].

Kushibiki et al. researched the efficacy of talaporphirin-mediated photodynamic therapy applied to cervical cancer cells. In this study, the levels of miR-210, an important regulator of cell metabolism in hypoxic conditions, were upregulated, along with miR-296, a small miRNA involved in a positive loop with vascular endothelial growth factor (VEGF). It is the authors’ opinion that the levels of these miRNAs could prove a predictive factor of therapy efficacy and a potential target for antagomir therapy [31]. The most recurrent miRNAs and their roles are summarized in Table 2.

## 4. Discussion

The aim of this review was to organize the current knowledge about light-based therapy and the correlation with miRNA expression in different skin conditions, ranging from infectious diseases and immune-mediated dermatoses up to tumoral and pre-tumoral conditions. Out of five studies dealing with miR-21, four either tried to suppress its effects and levels after the treatment or used it as a marker of disease activity, whilst only one assessed it as an enhancer of therapeutic effects. miR-21 was observed in several studies (here reviewed) as an anti-apoptotic factor and was related to a thicker Breslow level in the case of melanoma and to a poorer prognosis in the case of squamous cell carcinoma [32]. The role of mir-21 in the development of cancer is so well established that it was studied in different diseases such as lung cancer: in this case, the upregulation of miR-21 represented a pro-tumorigenic factor, inducing the RAF/MEK/ERK pathway, dedicated to the regulation of the cell cycle, and increasing the production of IL-8 [33]. miR-21 has been observed to drop its levels after adequate UVB therapy in the case of psoriasis [8]. It is interesting to note that studies regarding basal cell carcinoma (BCC) and miR-21 levels are still lacking and can prove an interesting field of research since miR-21 belongs to the survival cell process linked to p53, a well-known protein involved in the development of BCC [34]. In this regard, UVB therapy, and UV rays in general, may explain both the pathogenetic process of such tumor and also its low-grade invasiveness. Regarding PDT, this light-based treatment seems to block the effects and expression of miR-21, with pro-apoptotic results, thus suggesting its possible role as a routine adjuvant treatment even in cases of locally advanced tumors such as melanoma and SCC. The only study showing a possible use of miR-21 combined with PDT might also suggest a possible treatment for these skin conditions and tumors rather than just a diagnostic tool or a target therapy, for example, using antagomirs as anti-tumoral agents [33] as in melanoma. After surgery, chemotherapy and strict follow-up exams, both photodynamic therapy and sonodynamic therapy seem to be an interesting alternative, according to some studies [15]. Similar to this, a very recent study suggested a possible role of miR-21 as a marker of bodily response in vitiligo [34], hence proposing a new possible explanation of how phototherapy could lead to repigmentation of white areas via the upregulation of this particular miRNA. Still though, as of today, these protocols have not been validated yet and are far from being accepted as routine treatments.

Three studies researched the levels and role of miR-155 at the skin level. miR-155, out of all the miRNAs, seems to be one of the most consistently related to melanoma and lymphomas [35], and other skin-related processes such as fibrosis [36]. One of these three studies calculated the levels of miR-155 after a combined therapy with UVB irradiation and methotrexate administration. miR-155 acts at the level of keratinocytes, if expressed more than the normal levels, as a survival factor. Additionally, miR-155 seems to be involved in the polarization of T cells, either to a Th1 or a Th17 profile [37]. In another study, it was shown that miR-155 is overly expressed in the case of breast cancer and is subsequently reduced when the tumor size is decreased after treatment [14]. Its possible role as a marker of disease activity, potentially more sensitive than conventional tumoral markers such as carcinoembryonic antigen (CEA) and cancer antigen 125 (CA-125) along with miR-21 [14], has been suggested. One final study by Cao et al. used miR-155 as a target for the deliverance of nanoparticles loaded with anti-tumoral drugs to best deliver target therapies: this suggests to us that, in fact, miRNAs can be used not just as a diagnostic or therapeutic tool alone but also as eligible targets for theranostic procedures [24].

miR-145 seems to be another possible target for therapeutic and imaging purposes. In one study, after PDT treatment of oral cancer, the levels of miR-145 dropped significantly. In the same study, cells presenting with low levels of miR-145, after being transfected with miR-145 mimics, showed a better result after the treatment via the increase in apoptotic processes and inhibition of progression through the pathway of runt-related transcription factor 3 (RUNX3), an oncogene involved in head and neck cancers [17]. These results, however, were not achieved in another study: in this last case, although increased levels of miR-145 were linked to better treatment results, such levels did not decrease after treatment but rather increased [18]. Similar results were achieved in another study, where nanoparticles containing melanin were used to deliver the drug in situ [21]. These three studies, when compared, suggest to us that miR-145 is an miRNA that still requires further experimentation, or, at the very least, that the usage of either aminolevulinic acid or pheophorbide could provide considerably different results in the expression of this miRNA.

Another miRNA that seems of importance in the case of cutaneous cancers is miR-125. This miRNA targets signal transducer and activator of transcription 3 (STAT3), a gene well known for its implication in tumorigenesis via migration and invasion of malignant cells. In the case of skin cancers, particularly squamous cell carcinoma, higher levels of this miRNA seem to act as a protective factor by downregulating STAT3 [38]. In this review, the only study found linking this miRNA to some light-involving therapeutic tool regarded breast cancer [14], but it can be suggested to extend this treatment to other skin tumors where the pathway of STAT3 could be of pathogenetic significance. One more study [8] researched the role of narrowband UVB therapy and its effects on the expression of miR-125, showing an increase in its levels and, as a molecule targeting p53, a gain of function for this crucial protein in psoriatic lesions. Given the role of p53 as a “genome guardian”, its role in another important epithelial tumor such as basal cell carcinoma can be evaluated [39].

One final miRNA that has been investigated the most after light-related treatments in the case of skin diseases is miR-29. This small miRNA seems implicated in fibrosis-related processes, particularly its regulation of TGF-beta and the Akt signaling pathway [13]. In normal conditions, this miRNA’s levels are normal, allowing TGF-beta to solve its role as a collagen production promoter, particularly collagen I [13]. In another study regarding skin fibrosis only, higher levels of miR-29 were desirable to lower the rate of collagen deposition through high-fluency red light treatment [26]. Beneficial effects, in the case of skin aging and scleroderma, were achieved with a combined treatment of laser therapy and vitamin A administration [13]. Given the well-known role of the derivates of vitamin A, not just for skin fibrosis and aging but also for other diseases such as acne, the combined role of retinoids and laser therapy, particularly in the treatment of acne, and the relationship between the treatment efficacy and miRNA levels at the lesional skin would be interesting topics for research. Furthermore, since these two studies tried to achieve opposite effects by either upregulating or downregulating this miRNA, it is sensible to consider comparing different light-emitting sources and their effect on miRNA levels for each pathogenetic process.

The rest of the studies proposed a plethora of miRNAs involved at various stages of different transcriptional processes. The most recurrent ones were related to apoptosis and cell growth/replication or neo-angiogenesis, in some cases, related to onco-hematological diseases [27], but, to this day, their role in inflammation and skin-related diseases has been poorly researched. It is worth mentioning that these miRNAs could provide a target for future therapies that require a stop in angiogenesis or where there is inflammation after an originator. Regarding the topic of fibrosis, miR-21 along with miR-23 and miR-31 seems to be closely related to profibrotic processes [26]. miR-23 is related to a superfamily of miRNAs implemented in angiogenesis called “angioMIRs”. AngioMIRs have been found as positive or negative regulators of angiogenesis through inhibition of pro-angiogenetic pathways, including well-studied molecules such as VEGF, or inhibition of anti-angiogenetic factors (such as the HOX pathway, VECAM-1 and Ephrin) [40]. These miRNAs could provide, if regulated properly, a helpful tool in those disorders of the skin where endothelial dysfunction plays a major role, such as diabetic ulcers. Moreover, since photodynamic therapy has been used for the treatment of recalcitrant diabetic ulcers, it could prove useful considering the option of implementing miRNAs to produce faster and better results, possibly reducing the time of exposure or the required sittings.

Regarding psoriasis, most of the studies suggested the possible role of miRNAs as powerful tools against the disease’s progression, either as activity markers or as potential targets. PUVA therapy [10,11] and narrowband UVB therapy [9,28] regulate the keratinocyte cycles by upregulating or downregulating several miRNAs. It is worth noting that by silencing the anti-apoptotic miRNAs involved, these types of treatments could provide some relief to patients from the administration of certain drugs such as methotrexate or ciclosporin. These two drugs, even though very well known as effective treatments for psoriasis, in fact, present several side effects that can prove to be a limitation for their administration.

Finally, it is worth mentioning that the role of miRNAs in several other skin diseases has yet to be researched properly [29]. Several miRNAs are related to different cell populations, cell types and stages of the same disease. On the other hand, diseases such as skin tumors are involved in several physiological and pathological processes, such as vascularization, immune response and oxidative stress, all of which are potential targets for light-based therapies such as PDT [41]. In the case of melanoma, oxidative stress acts as an anti-tumorigenic stimulus if levels of ROS are high enough to trigger apoptosis and inhibit cell migration [42]. Consequently, achieving higher ROS levels by either inhibiting or promoting the miRNAs linked to the stress response can be suggested for future therapies. The results of the above-mentioned articles lead to the conclusion that, given the complex regulatory mechanisms involved at a nuclear or post-nuclear level, these molecules could prove to be a very important hint to treat systemic diseases possibly with light-related treatments and inhibiting molecules such as antagomirs but can also prove to be a confounding factor for different diseases coexisting in the same patient, providing different or unmatching miRNA levels when measured.

**Figure 1:**
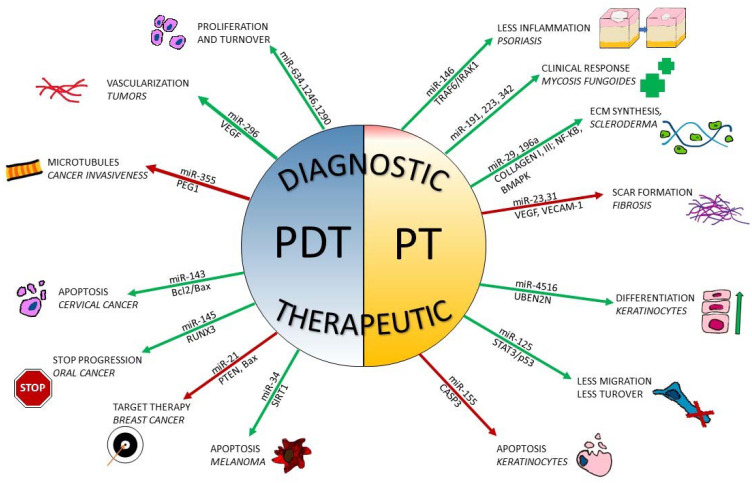
Effects of photodynamic therapy/phototherapy on miRNA expression in skin conditions. Green arrows represent upregulation of circulating/local levels of miRNAs; red arrows represent downregulated levels of miRNAs. On the upper part of the figure, miRNAs are exploited as a diagnostic tool; on the lower part, miRNAs provide therapeutic targets or produce healing effects when down- or upregulated.

The conclusions provided here are summarized in Figure 1.

## 5. Conclusions

Since their first applications, light-based therapies have helped several skin conditions, both related and unrelated to malignancy. Being both secure and scarcely invasive, these treatments have proven to have generally scarce side effects, but the current knowledge on their mechanism of action is lacking. To better understand the molecular pathways involved in the healing process, miRNAs have proven to be a helpful tool both as a diagnostic marker and as a possible target therapy. Either by targeting miRNAs or using mimics/inhibitors, the effects of light-based therapy can be enhanced or predict treatment efficacy. Current studies are not enough to focus on just one treatment, but the premiere results are promising. According to the results provided here, certain miRNAs appear to be more frequently associated than others in chronic skin conditions, being either up- or downregulated, and it can be suggested that further clinical trials might focus on targeting or exploiting these molecules.

## Figures and Tables

**Table 1:** References of miRNAs and the cell line or pathophysiological process studied. Increased miRNAs are shown on the left, and decreased miRNAs are shown on the right. The table was divided into two parts according to the type of treatment considered: phototherapy, laser therapy, PUVA, photoacoustic therapy (up) and PDT (down). Articles in which miRNAs were used only for diagnostic purposes are marked by an asterisk.

Authors	Increased miRNA	Decreased miRNA	Target
Phototherapy, laser therapy, PUVA, photoacoustic therapy			
Zhang et al.	206		fibroblasts
Zhang et al.	21		HeLa cells
Fan et al.	145-5p		laringeal cancer
Mamalis et al. *	29, 196a	21, 23b, 31	fibrosis
Qu et al.		29a	skin rejuvination
Chowdhari et al.	4516		psoriasis
Chowdhari et al.	4516		psoriasis
Mcgirt et al. *	191, 223, 342		T cell lymphoma
Gu et al.	125b	21	psoriasis
Wang et al.	193		mesenchymal cells
Ele-refaei et al. *	146a		psoriasis
Soonthornchai et al.		155	psoriasis
Khori et al.	125a	155, 21, 376b	breast cancer
PDT			
Kushibiki et al. *	210, 296		HeLa cells
Bach et al. *	487b, 634, 1246, 1290		tumoral cells
Hu et al.	34a		melanoma
Li et al. *		355	tumor cells
Guo et al.	143		cervical cancer
Moon et al.	9-5p, 192-5p, 193a-5p	32-5p, 143-5p, 145-5p	oral cancer
Jin et al.		34a	cervical cancer
Fang et al.	145		oral cancer
Wu et al.		21	breast cancer
Assali et al.	101		breast cancer
Cao et al.	155		target therapy
Qian et al.		21	target therapy

**Table 2:** Main miRNAs resulting from research correlated with light-based treatments. From left to right: miRNA involved, main pathways, effects if up- or downregulated, other related conditions.

miR	Pathways Involved	Effects If Decreased	Effects If Increased	Conditions
21	Akt, PTEN, Bcl2/Bax, RAF/MEK/ERK p53	Cellular differentiation	Carcinogenesis, immune cell activation	Lung cancer, skin aging, melanoma, basal cell carcinoma
29	TGF-beta, Akt, COL1A1, COL1A2, COL3A1 and FBN1	Upregulation of collagen and fibrillin deposition; tumor-suppressing properties	Negative regulator of collagen expression	Fibrosis-related processes, skin aging, sclerodermia, melanoma, liver, colon, cervical and lung cancer
125	STAT3; p53	Cell migration, cell turnover	Apoptosis, cell killing	Breast cancer Basal cell carcinoma
145	RUNX3	Cell migration and proliferation	Increase in apoptotic processes, inhibition of tumor progression	Oral cancer, diabetes
plese155	CASP3, NF-kB, SOCS1	Inflammation inhibition, less tumoral growth, activation of Th2 pathway	Survival factor, cell polarization	Melanoma Lymphomas Skin fibrosis Breast cancer
23 31	VEGF; HOX; VECAM-1; Ephrin	Lesser collagen deposition	Fibrosis, angiogenesis	Inflammatory bowel diseases, tumors, skin wound healing
